# A Lip Repositioning Technique Using Polyester Threads for Gummy Smile Treatment

**DOI:** 10.1155/2022/3972150

**Published:** 2022-10-15

**Authors:** Renata Oliveira Ribeiro Horn, Carlos Nelson Elias, Júlio César Joly

**Affiliations:** ^1^São Leopoldo Mandic University, Campinas, SP, Brazil; ^2^Instituto Militar de Engenharia, Rio de Janeiro, RJ, Brazil

## Abstract

A new technique is proposed in this study to correct the gummy smile (GS) with myotomy, combining lip repositioning with the insertion of polyester threads at the surgical site to act as a physical barrier and control relapse. 11 patients were clinically assessed (30.2 ± 7.43 years old, 90.9% females and 9.10% males). All patients presented gingival display (GD) greater than 4 mm. Hypermobile upper lip (HUL), vertical maxillary excess (VME) + HUL, altered passive eruption (APE) + HUL, and VME + APE were the etiologies identified. Three polyester threads were inserted in each patient one month after the surgery. The GS was measured before, 6 months, and 12 months after the surgery. The results showed a reduction in the mean GD of the patients, 4.42 mm after 6 months (*p* value = 0.000) and 4.13 mm after 12 months (*p* value = 0.000). The largest relapse was 0.29 mm and was not statistically significant (*p* value = 0.07). The Friedman test with pairwise comparisons was used to determine the existence of statistically significant differences in GD between the periods analyzed. The results showed that the proposed technique was successful in treating GS, presenting significant reductions in the GD 12 months after surgery and controlling the relapse.

## 1. Introduction

Smile aesthetics is directly related to the patient's emotional state. Dentists and laypeople perceive a gummy smile (GS) as a nonaesthetic factor [1–3]. Furthermore, it is worth saying that 14% of women and 7% of men present that characteristic [4]. GS smile is characterized by an exposition of the gingiva greater than 3 mm during the smile, measured from the zenith to the upper lip [3]. This condition may be associated with several etiological factors, among them altered passive eruption, vertical maxillary excess, gingival hyperplasia, muscle hypermobility, and short upper lip. Patients with GS may present more than one etiological factor and require an accurate diagnosis for proper treatment [5–8].

Lip repositioning is an alternative for GS treatment in case of vertical growth of the maxilla from mild to moderate and hypermobile lip [9–11]. This technique aims to reduce the depth of the upper vestibule, limiting the tensile stress of the muscles involved in the smile by removing a mucous strip from the bottom of the vestibule and suturing the lip mucous to the mucogingival junction [12–14]. Furthermore, this surgery is an effective approach, especially in cases with small discrepancies, and is less invasive than orthognathic surgery, with a more immediate result when compared to orthodontics and longer lasting than the application of botulinum toxin. The use of hyaluronic acid is also considered an approach to GS treatment, being an alternative to more invasive approaches [15].

Currently, there are six modified techniques of lip repositioning [16]. The lip repositioning technique with muscle rupture (myotomy) is more successful than the technique without myotomy when compared [10, 16–20]. However, among all the modified lip repositioning techniques that existed, the one with periosteal suturing presented the greatest reduction in the gingival display of the patients; nevertheless, relapse is still recurrent [16, 19].

The insertion of biomaterials acting as barriers can be a way to prevent muscle reinsertion. The literature shows that the use of polyester sutures in periodontal surgery has positive results, including compatibility with oral tissues, and might be a beneficial alternative to avoid muscle shape memory [21–24].

The purpose of this work is to present a new approach to GS treatment regarding the control of relapse. It is worth saying that it is not part of the scope of this study to prove the efficiency of the proposed technique, being necessary for the elaboration of a deeper study, including a control group.

The proposed technique consists of the insertion of polyester threads, horizontally, in the region lateral to the frenum, through a minimally invasive way, in the depth of the bone, depositing it in the subnasal region and the nasal fossae to prevent the new reinsertion of the zygomaticus minor muscle. Polyester multifilament suture thread was chosen as the filling material. This suture thread is a nonabsorbable synthetic material and is classified as an inert biomaterial. Inert biomaterials, when inserted into the body, induce a fibrous tissue capsule formation [25–28].

## 2. Materials and Methods

### 2.1. Study Design and Procedures

This study reports a series of cases of 11 patients that were selected and treated at a private clinic in Niteroi, Rio de Janeiro, Brazil, from January 29th, 2019, to August 14th, 2020. This research was approved by the Ethics and Research Committee of the Dental School São Leopoldo Mandic (Campinas, Brazil), as a number: 4.414.717, before the initiation of the trial, which was registered with the Brazilian Clinical Trials Registry (REBEC Reg No. RBR-79fbccw).

Patients of both genders, seeking a GS treatment, with a minimum age of 18 years, minimum gingival display of 4 mm from zenith to the bottom part of the upper lip during the smile, and systemically healthy and capable of maintaining good oral hygiene, were invited to participate in the research. Lip repositioning surgery was performed on 11 patients, one man and ten women. This sample was based on previous studies described in the literature about clinical cases of surgery of lip repositioning and myotomy [29–31].

Patients in states of pregnancy or lactation, smoking, alcoholism, systemic health problems, underuse of medications that could interfere in the process of healing, and periodontal disease active were excluded from the sample. The used criteria and the patient's health history and anamnesis were clinically and radiographically assessed to be included in the study. All patients were treated at Renata Horn's Dental Clinic. The participants were submitted to a complete dental exam to test their oral health and were forwarded to clinical treatment, including standard periodontal therapy, being also instructed about oral hygiene.

**Figure 1:**
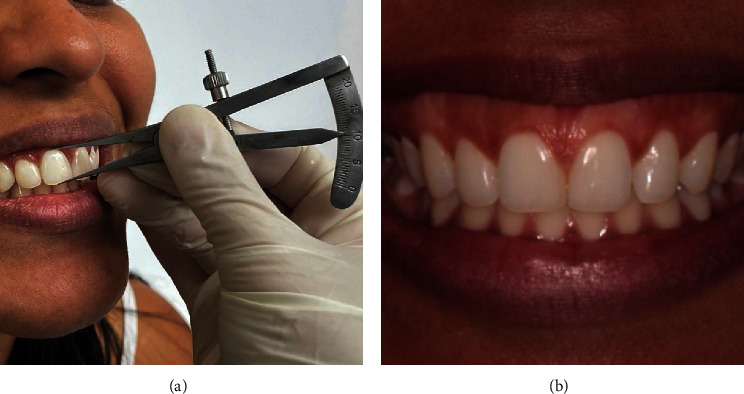
Patient's picture before surgery. (a) Measurement of the length of the patient's upper right central incisor. (b) Patient's picture at the maximum smile.

Participants of the study were assessed previously and 6 and 12 months after surgery. Patients had the maxillary central right incisor by using a spectrometer (Figure 1(a)), and in addition, photographs were taken (1 : 1), while the patient was smiling maximally (Figure 1(b)). Afterward, the photos were analyzed by using Keynote v.12.1, and the diagnosis of each patient was determined through measurements by using a computer ruler, calibrated from the length of the right central incisors, collected at clinical screening.

**Figure 2:**
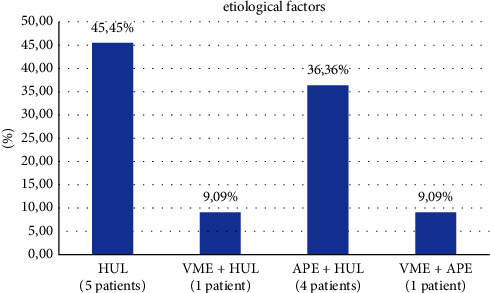
Representative graph of etiological factors identified in the sample group.

Patients were then diagnosed with one or more etiologies (Figure 2). To identify HUL, patients had their upper lip mobility measured from rest to maximum smile by using a ruler. Patients who had upper lip mobility greater than 8 mm were diagnosed with HUL [32–34]. APE was determined in patients who had the four maxillary incisor teeth short or quadratic in aspect (length/width ratio equal or greater than 0.85) when the incisal wear was absent (tooth wear index equal or lesser than 1) [35, 36]. VME, on the other hand, was detected in patients who had the length of the lower third of their face greater than the other two-thirds [37].

### 2.2. Surgical Procedure

The patients were instructed to rinse their mouths with chlorhexidine 0.12% previous to the surgical procedure. After anesthetic administration by infraorbital and infiltrative injection (2% mepivacaine with 1 : 100,000 epinephrine), the demarcation of the parallel incision lines was performed. Besides lip repositioning, crown lengthening was performed in patients diagnosed with APE associated with an HUL [37]. Patients with APE were diagnosed by using probing, while the surgery was performed through the measures acquired based on the cement-enamel junction. The planning of each patient was considered in a way that the base for the crown lengthening to the patients that would receive restoration was the dental smile designer (DSD).

Patients who had 3 mm of attached gingiva after lip repositioning received crown lengthening in the same surgical act, and patients who had less than 3 mm of attached gingiva received the crown lengthening previous to lip repositioning.

For lip repositioning, the first incision began by the region of the frenum, for easy access, following the mucogingival line contour, as proposed in previous studies [13, 14, 30]. The second most apical incision was performed considering the distance of twice the gingival exposure relative to the papilla. The average distance of the exposition varied between 8 and 15 mm [5]. The incision was carried out by tilting the 15C blade at 45° (Swann-Morton Ltd., Sheffield, England). This angle of incision allowed the preservation of the periosteum, enabling the internal suture of the muscle.

**Figure 3:**
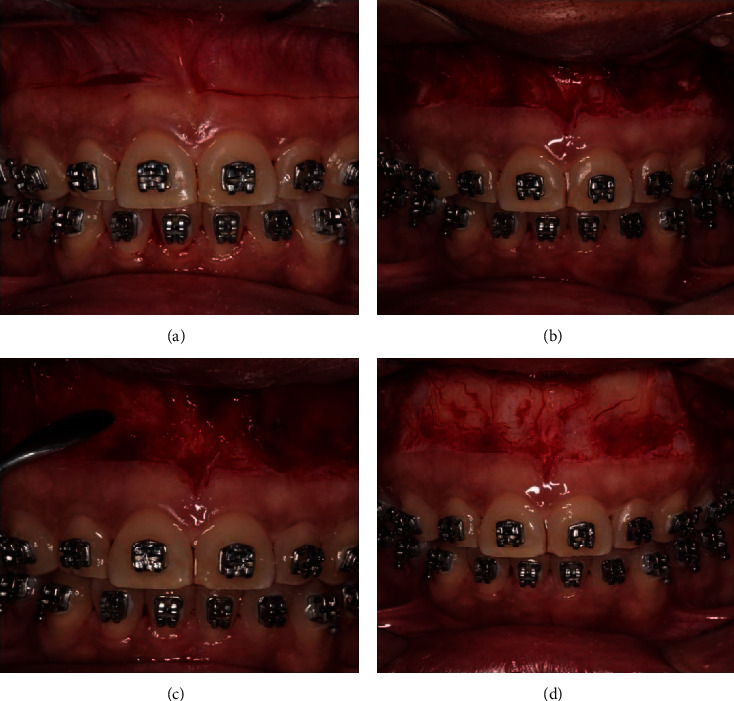
(a) Demarcation of the incision lines. (b) Tissue remotion. (c) Muscle detachment in the direction of its insertion origin. (d) Muscle away maintaining a periosteum band for the suture.

Based on some authors [17, 18, 30], the horizontal incisions were extended to the area of the second premolars or first molars, depending on the width of the smile exposition, and vertical complementary incisions were performed at the distal limits, bilaterally, for the union of horizontal incisions. After this phase, the region which was cut was divided for complete tissue removal of 1 mm of thickness on average. The detachment of the muscle fibers was carried out by using a raspatory scalpel (Welfare, Germany), following the papilla on of the insertion origin. This removal aimed to increase tissue stability (Figures 3(a)–3(d)).

**Figure 4:**
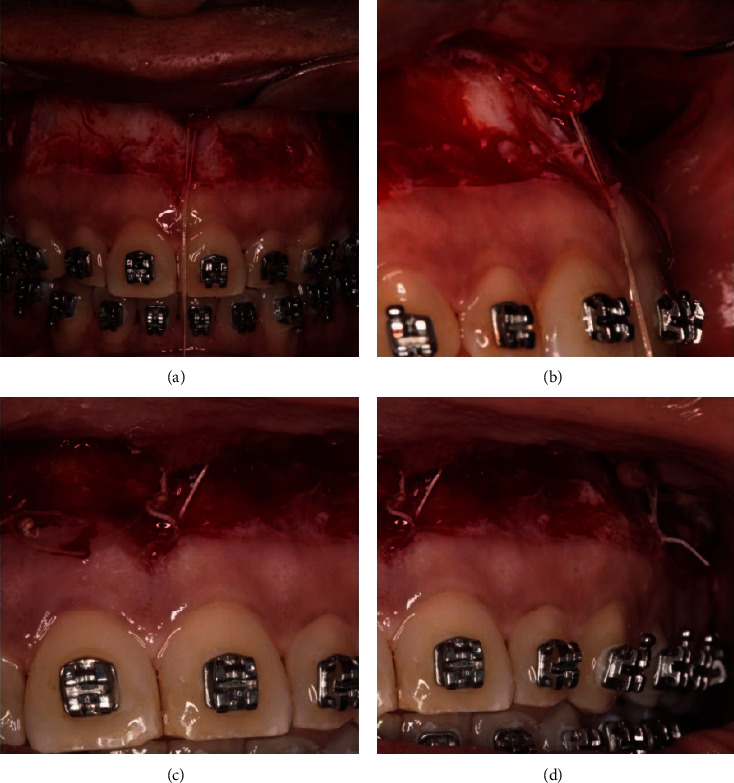
First suture with polyglactin at the medium line: (a) front view and (b) side view. Muscle stabilization: (c) front part and (d) back part.

A muscle suture was performed in the region of the frenum, accompanied by an internal suture on each side in the region between canine and premolar, besides a continuous suture containing the muscle previously sectioned, with 4-0 polyglactin thread (Vicryl Ethicon, Guaynabo, Puerto Rico) (Figures 4(a)–4(d)). The external sutures were performed in two stages, including main sutures, by using 5–0 nylon thread (Vicryl Ethicon Co., Guaynabo, Puerto Rico) at the height of the papillae, beginning by the midline, to maintain an anatomic orientation [17].

**Figure 5:**
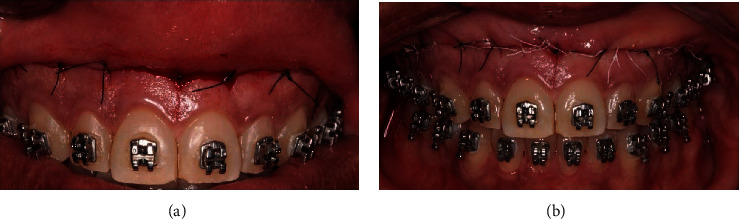
(a) Main external sutures at the height of the center of the papilla. (b) Complementary sutures among the main sutures.

Furthermore, complementary sutures were performed with 6-0 PTFE thread (KeyDent Co., Saudi Arabia) between the main sutures (Figures 5(a) and 5(b)), aiming to obtain a thorough primary closure of the surgical wound. Postoperatively, a cold compress was recommended immediately after surgery and minimal lip movement for 15 days. Patients were prescribed an anti-inflammatory (Arflex, 200 mg) once a day for 3 days, an antibiotic (amoxicillin, 500 mg) every 8 hours for 7 days, and an antiseptic mouthwash (chlorhexidine) twice a day for 10 days.

Insertion of polyester thread 3-0 named Bioline (Bioline Fios Cirurgicos Ltda, Annapolis, Brazil) was performed by injection one month after the surgery to respect the period of full healing of external tissues and because, according to some studies [10, 18], the relapse starts to be significant from the third month after the surgery.

**Figure 6:**
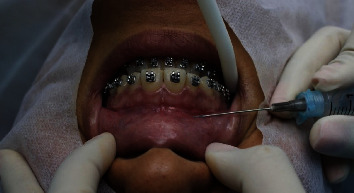
Insertion of polyester thread 3-0 near the subnasal region.

The material was put inside a 3 mL syringe with a 26 G ½ (0.45 × 13 mm) needle. The procedure was carried out with the insertion of the thread laterally to the upper lip frenum through a technique (less invasive than orthognathic surgery and more durable than botulinum toxin), with back and forth movements, injecting the thread horizontally at a depth of the bone. The polyester thread was deposited at the subnasal region by using lateral elements as a reference for the needle movements, thus aiming for the prevention of the reinsertion of the nose wing levator muscle and the upper lip. The thread was also inserted in each nasal cavity, using the canine and second premolar as reference for the needle movements, to prevent the new reinsertion of the zygomaticus minor muscle, acting as a physical barrier (Figure 6). Three polyester threads were inserted in each patient.

### 2.3. Thread Characterization

The thread morphology was observed by using the scanning electron microscope (SEM) FEI Quanta FEG 250 (Field Emission Gun FEI Quanta FEG 250, Hillsboro, Oregon, USA), which was adjusted according to the individual character of each sample.

### 2.4. Statistical Analysis

A satisfaction survey was conducted with all patients before and after one year of the surgical procedure. Based on individual experience with the surgery and results, each patient assessed if they would recommend the surgery to someone with gummy smile and if the lip repositioning technique applied promoted some benefits beyond the aesthetic.

Statistical analysis was performed by using SPSS v.23 (IBM Corp., Armonk USA), and data of gingival display of all patients, which were collected in the mentioned periods, were analyzed by using descriptive statistics.

Since the sample size was too small (less than 30), the Friedman test was used to verify the existence of statistical difference (*p* value <0.05) among measures of gingival display of patients, comparing the periods analyzed (baseline and 6 and 12 months postsurgery). For the statistical analysis, the null hypothesis was that there was no difference in the means of gingival display. The baseline was the control group. It also performed pairwise comparisons between the periods, aiming to verify in which specific period there were significant differences in the means of gingival display. Regarding the boundary conditions of the statistical tests, it can be said that the confidence interval and significance level considered were 95% and 5%, respectively.

## 3. Results

**Figure 7:**
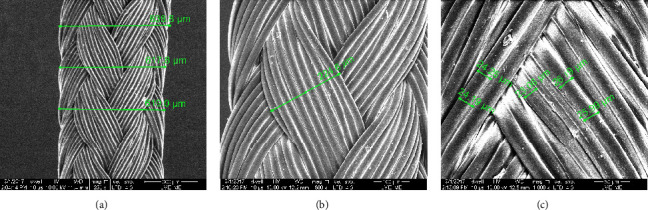
Morphologies of used thread at small magnification (a), wire rope (b), and thin wires (c).

Figure 7 shows the used threads' morphologies. It is possible to observe that the thread has a diameter equal to 620 *µ*m and is like a thread rope with strands of thin threads. Ten thin threads are twisted to form a strand with a width of 220 *µ*m. Each thin thread has a diameter close to 20 *µ*m.

During the entire postoperative evaluation period, all cases evolved uneventfully. Each patient had one or more etiological factors. Hypermobile upper lip (HUL) alone was the etiologic factor present in 45.45% of the cases. The other cases analyzed were a combination of etiological factors such as vertical maxillary excess (VME) with HUL (9.09%), altered passive eruption (APE) with HUL (36.36%), and VME with APE (9.09%) (Figure 2).

**Figure 8:**
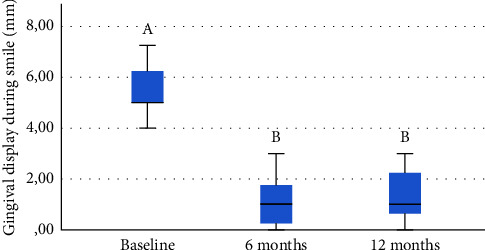
Boxplot of the gingival exposure for baseline and 6 and 12 months after treatment.

**Figure 9:**
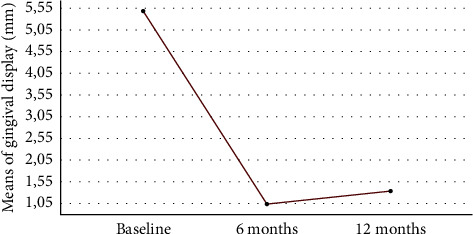
Mean of gingival display along the periods analyzed.

Before surgery, the patients had a mean gingival display when smiling of 5.48 ± 0.98 mm. Clinical follow-up showed the exposure of 1.04 ± 0.99 mm after 6 months and 1.34 ± 1.04 mm after 12 months. From baseline to 6 months, the reduction was 75.6%, and from baseline to 12 months, the reduction was 75.5%. From 6 months to 12 months, there was an average increase of 0.29 mm, which was not a considerable difference, indicating thus stability in the results (Figures 8 and 9).  Table 1 shows a summary of data from the research, which includes the age of patients, their etiologies, and measurements of gingival exposure from baseline, 6 months, and 12 months.

The result of the Friedman test shows that there is a significant difference in the means of gingival display of patients (*p* value ≤0.000), as it is presented in Table 2. Performing pairwise comparisons between the periods analyzed, it was found that the periods from baseline to 6 months and from baseline to 12 months showed significant differences (*p* value <0.05) in the means of gingival display of patients. Moreover, the period from 6 months to 12 months after surgery showed no significant difference in the means of gingival display (*p* value >0.05), which indicates that the relapse control was successful (Tables 2 and 3). These results showed that the surgery decreases the gingival display and maintains stability until 12 months after surgery.

**Figure 10:**
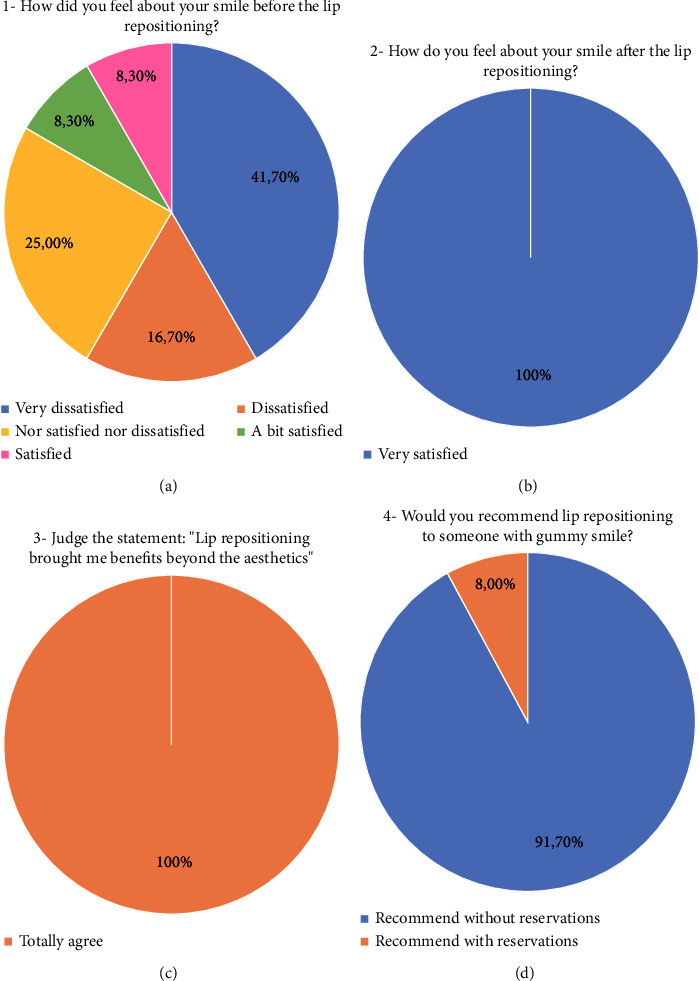
Answer of the question carried out for the evaluation of patient satisfaction, serving as an auxiliary analysis instrument. (a) Satisfaction graph of patients before the surgery. (b) Satisfaction graph of patients after the surgery. (c) Satisfaction graph of patients with the new condition reached. (d) Graph of recommendation degree of lip repositioning to someone else.

In a complementary manner, an opinion questionnaire was carried out for the evaluation of patient satisfaction, serving as an auxiliary analysis instrument. The questions were elaborated based on the Likert scale, taking into consideration a response pattern outlined by a psychological metric (psychometric scale). The results of the survey are shown in Figure 10. The results showed that prior to treatment, most patients (58.4%) were very dissatisfied or dissatisfied with their smile (Figure 10(a)) and that one year after treatment, all patients (100.00%) were satisfied (Figure 10(b)), even claiming benefits beyond aesthetics (100%) (Figure 10(c)) and that they would recommend the procedure to other patients (91.7%) (Figure 10(d)).

## 4. Discussion

Inspired by the randomized study of the modified techniques of lip repositioning with and without myotomy [10], this research adopted the technique with myotomy since it promotes better results in terms of stability [14, 16, 20]. Some authors ratify this information by showing that the results of modified lip repositioning surgery remained stable for up to six months postoperatively, presenting only a few cases with relapse. The same related this fact to muscle memory, trying to restore its activity [14, 19].

Knowing that the lip repositioning technique, being classic or modified, has the objective of limiting muscle traction, reducing the deep of the upper vestibule with an expected average improvement of 3.4 mm ± 0.4 mm of GD [14], the results found in this study presented an average reduction of 4.42 mm in 6 months and 4.13 mm in 12 months (Table 3), which corroborate with the outcomes found with the application of the modified technique [16, 18, 30, 38, 39]. Likewise, literature shows that lip repositioning by using the classical technique is also able to achieve good results, with an average of 4.5 mm reduction in 6 months [16, 32].

It is worth mentioning that the results obtained with the proposed technique had a high degree of satisfaction (Figure 10), which was similarly recorded by the patients of other studies by using other techniques of lip repositioning [16, 20].

The satisfaction questionnaire applied among the patients in this study showed that 41.7% of the patients before treatment were very dissatisfied with their smiles (Figure 10(a)), suggesting that excessive gingival exposure can affect the well-being of individuals. From this, it can be understood that this technique contributed not only to the aesthetic adequacy of patients but also to the improvement of factors related to self-esteem. After the clinical procedure, all patients stated that they felt very satisfied. This result allows us to say that the benefits achieved provided greater quality of life and comfort to the individuals affected by the gummy smile.

Moreover, all patients would recommend the surgical lip repositioning treatment, 91.7% of them reporting that they would do it without reservations, showing good acceptance of the proposed technique. Finally, 100% of the patients reported benefits beyond the aesthetic with the treatment performed. This was also applied to questionnaires to patients by some authors [30], and the results showed great acceptance and satisfaction by patients with the treatment performed. This result corroborates the findings of other authors [14, 32]. Nevertheless, one study [14] pointed out that the patients were satisfied with the results presented by both lip repositioning techniques, with or without myotomy.

Although recent studies have pointed out the modified technique of lip repositioning with periosteal suture as the one with greater reduction in GD, its stability is not related to the degree of reduction achieved.

It is known, though, that the relapse is expected if of 25% to the lip repositioning surgery [20]. That occurs due to the action of the muscle responsible for the upper lip elevation while smiling, in other words, levator labii superioris alaeque nasi, levator labii superioris, and zygomaticus minor, due to its high capacity of regeneration [40].

According to the literature [18], some studies lost 100% of their results with the use of the classical technique, maintaining the labial frenum, as proposed by some authors [32], and not reaching the muscle, as proposed by other authors [5].

The presented technique aims to ally myotomy to a physical barrier, with the objective of stabilizing the achieved result in lip repositioning. This proposal is based on studies performed with hyaluronic acid to GS treatment in medicine, where the referred acts as a physical barrier compressing the lateral fibers of the muscle levator labii superioris alaeque nasi, containing the mobility of the deeper portion of it and mitigating the movement of the upper lip during smiling. In that way, the threads also tend to act similarly. For that reason, they were applied in the same anatomic region approached in some studies [15, 41].

Figure 7 shows the polyester thread used in the present work, being of the type tressed. Its benefits include greater flexibility and handling in terms of the performance of movements such as turn, bend, and twist, being in that way preferable to surgeries in small spaces. Furthermore, it is noteworthy that multifilament threads have a structure like the collagen arrangement of muscles. Collagen fibers are able to join the surface of the thread and form a volume of fibered tissue that fills and compress the muscle fibers, likewise the hyaluronic acid [15, 41, 42].

**Figure 11:**
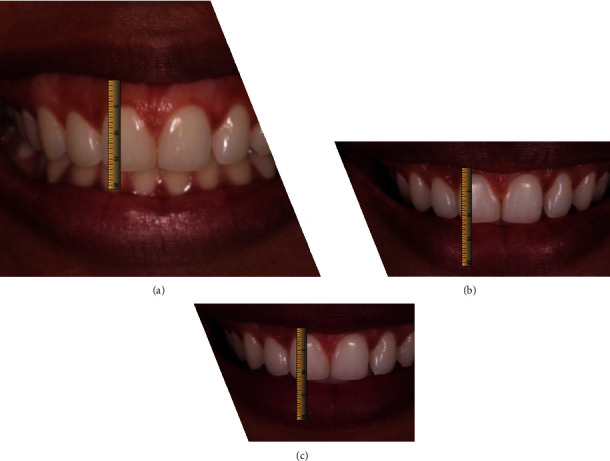
(a) Measurement of an initial smile. (b) 6 months postsurgery. (c) 12 months postsurgery.

However, unlike these authors, this study extended the follow-up period to 12 months, showing a reduction of 4.13 mm (75%), a value higher than that found in the technique with myotomy in a shorter period of 6 months postoperatively [18]. These results are relevant since that relapse tends to intensify within 6 months [14]. Clinical results can be observed from the photos of patients (Figures 11(a)–11(c)).

Reaffirming the importance of longer follow-ups, some authors [20] performed a systematic review in which the results showed that three months postoperatively, there can be a 2.87 mm reduction in the GS. After six months, these results had a slight decrease, with a 2.71 mm reduction in the condition, evaluating the techniques with and without myotomy. However, after 12 months of follow-up, the reduction in the gummy smile was 2.10 mm, indicating a substantial amount of relapse. Despite the importance of follow-up beyond six months, few articles evaluated patients after this period, and only four of the seven articles were included in the systematic review. In addition, the meta-analysis also showed a significantly greater reduction in a gummy smile when the technique included myotomy.

That said, regarding the stability promoted with the use of polyester thread, the results showed that the technique proposed could be considered a new alternative for the treatment of the etiologies HUL and VME from light to moderate. HUL was the etiological factor presented in most of the cases (45.45%), followed by the combination of APE with HUL (36.36%). This agrees with the findings of one study [11], in which most of the cases studied (45.3%) had HUL alone and 34% had an association between HUL and APE.

As shown in the Materials and Methods section (Figure 2), 5 patients had APE combined with another etiological factor; besides, as was said previously, these patients received the modality of treatment of lip repositioning and crown lengthening. 12 months after the surgery, the gingival display of these patients was measured again, and the average reduction achieved from the lip repositioning technique was 2.55 mm (55%), whereas the average reduction from the crown lengthening technique was 2.10 mm (45%). Then, the average reduction achieved from the combination of these two techniques was 4.65 mm.

In cases involving HUL alone and HUL with VME, lip repositioning was the technique used in the treatment, as indicated by some authors [30, 43]. In cases involving APE with HUL and APE with VME, lip repositioning was used associated with crown lengthening, as indicated in one study to treat APE to achieve better results [20, 42]. Furthermore, new studies have pointed out that lip repositioning associated with other approaches, such as crown lengthening, restorative procedures, or injections of botulinum toxin, tends to be beneficial to more stable and accurate results.

It is important to quote that some authors also kept the follow-up of patients for at least 12 months [17, 30, 31]. Conducting the study for more than six months is important to describe the actual condition of the results after surgery and long-term stability, as there are nonsurgical methods, such as botulinum toxin, that last for 3–6 months without causing structural and psychological damage to surgery.

From the results obtained in the present study, it was possible to observe that the proposed treatment was successful in managing gingiva exposure when smiling and in the maintenance of the results in a follow-up period of at least 12 months.

### 4.1. Limitations and Suggestions

The results presented must be interpreted cautiously considering some limitations such as the lack of a control group, small sample, lack of sample size calculations, absence of a randomized study, and longer follow-up periods. Based on these limitations, we encourage other authors to improve the research using a control group, the increasement of the sample size, sample size calculations, elaboration of a randomized study, and longer follow-ups. Also, we encourage authors to develop standardize measurements and forms of measurement and test the technique with other operators to benchmark the myotomy technique with polyester thread insertion.

## 5. Conclusion

Based on clinical results and respecting the methodological limitations of the present study, it is possible to conclude that the proposed surgical technique of lip repositioning with myotomy associated with the insertion of polyester thread as a physical barrier to limit muscle movement presented to be successful in the correction of GS during one year of follow-up. It was possible to successfully treat all patients who participated in this study. Randomized studies with longitudinal follow-up are needed to confirm the absence of relapse.

## Figures and Tables

**Table 1:** Data, means, and standard deviation of gingival display before the surgery and 6 and 12 months after surgery.

Patient	Age	Etiology	Baseline (mm)	6 months (mm)	12 months (mm)
1	40	HUL	6.5	2	2.5
2	27	HUL	4.5	3	3
3	43	HUL	5	1.5	2
4	26	HUL	5	1	1
5	35	VME	7.2	2	2.5
6	32	APE + HUL	5	0	0.75
7	28	APE + HUL	6.5	0	0
8	23	APE + HUL	5	1	1
9	34	APE + HUL	4	0	0
10	26	VME + APE	6	0.5	1.5
11	18	HUL	5.5	0.5	0.5
Means of gingival display			5.47	1.04	1.34
Standard deviation			0.97	0.98	1.04

HUL, hypermobile upper lip; VME, vertical maxillary excess; APE, altered passive eruption.

**Table 2:** Friedman test result.

*N*	11
Chi-squared	20.421
Degrees of freedom	2
*P* value	≤0.001

**Table 3:** Pairwise comparisons in the Friedman test.

	Means difference	Adjusted significance level (*p* value)
Baseline–6 months	4.42	≤0.001
Baseline–12 months	4.13	0.009
6 months–12 months	0.30	0.859

## Data Availability

The data used to support the findings of this study are openly available in Open Science Framework at https://osf.io/nrqwg/.

## Additional Points

*Clinical Significance*. Promote the correction of a gummy smile through a minimally invasive surgical procedure, with a high level of aesthetic acceptance by the patients and with biological stability.
